# Clinical evidence and mechanistic pathways of human milk oligosaccharide supplementation for health benefits: an updated review

**DOI:** 10.3389/fnut.2025.1599678

**Published:** 2025-07-07

**Authors:** Tanjina Amin, Md Mahmudul Amin, Adikari Arachchige Dilki Indrachapa Adikari, Xiaoming Zheng, Yibing Ning, Bing Wang

**Affiliations:** ^1^Gulbali Institute, School of Agricultural, Environmental, and Veterinary Sciences, Charles Sturt University, Wagga Wagga, NSW, Australia; ^2^Department of Anatomy and Histology, Faculty of Veterinary Medicine and Animal Science, Gazipur Agricultural University (formerly Bangabandhu Sheikh Mujibur Rahman Agricultural University), Gazipur, Bangladesh; ^3^Nutrition Research Institute, Junlebao Dairy Group Co., Ltd, Shijiazhuang, China

**Keywords:** human milk oligosaccharides, infant formula, gut-microbiota, infection, mechanistic pathway

## Abstract

Human milk oligosaccharides (HMOs), a diverse group of complex sugars, are increasingly recognized for their health advantages for infants. These bioactive molecules are believed to be critical in shaping gut microbiota, infant immunity, and overall health. Recent clinical studies have focused on supplementation of infant formulas with manufactured HMOs to replicate some of the benefits observed in breastfed infants. This review aims to summarize the latest evidence from human clinical trials on manufactured HMO supplementation, highlighting its associated health benefits and the underlying mechanisms of action. A comprehensive literature search was conducted using PubMed, Medline, and Scopus databases from 1964 to 2024, identifying clinical intervention studies on manufactured HMOs across different populations, ranging from pre-term infants to adults with or without medical conditions. Findings reveal that manufactured HMOs are safe, well-tolerated, and show promising benefits for immune health and gut microbiota composition, closely mirroring the effects of natural HMOs found in breast milk. Although studies have explored the prebiotic role of HMOs in modulating neuroactive metabolites such as short-chain fatty acids (SCFAs) produced by gut microbiota, there is a notable lack of research directly evaluating the cognitive outcomes of HMOs using MRI or standardized developmental assessment tools. Furthermore, this review highlights two novel clinical findings: the potential therapeutic role of HMOs in obesity prevention by promoting fat loss while preserving muscle mass and their beneficial effects in osteoarthritis by reducing pain and enhancing mobility. However, the variability in dosage, participant groups, intervention duration, and outcomes, along with the limited studies on the mechanistic pathways of HMOs, makes it difficult to draw definitive conclusions, underscoring the need for well-designed clinical trials across diverse health conditions to better understand the full potential of HMO supplementation.

## Introduction

Human milk oligosaccharides (HMOs) are key complex carbohydrates of breast milk, ranking as the third most abundant solid constituent after lactose and lipids (1, 2). Their concentration ranges from 12.9 g/L in mature milk to 20.9 g/L in early postpartum milk (3). This represents a ~ 100-fold higher concentration than what was found in bovine milk or infant formulas (4, 5). Over 200 distinct types of HMOs have been characterized, with approximately 70–75% being neutral HMOs, which include both fucosylated and non-fucosylated, and 10–30% being sialylated HMOs, distinguished by the presence of a sialic acid molecule at the terminal position (4, 5). HMOs play a crucial role in infant health, although they are not directly digestible by humans. Instead, they serve as prebiotics, fostering the growth of beneficial bacteria, particularly *Bifidobacteria*, in the infant’s gut, modulating the immune system, and supporting infant growth and cognitive development.

One of the major compositional differences between breast milk and infant formula is the presence of HMOs (6). Recent advancements in biotechnology have made it possible to produce HMOs identical to those found in human milk through bacterial fermentation (7, 8) and enzymatic conversion (9). As a result, specific HMOs such as 2′-fucosyllactose (2’-FL), 3′-fucosyllactose (3’-FL), Lacto-N-neotetraose (LNnT), 3’-Sialyllactose (3’-SL), and 6’-Sialyllactose (6’-SL) are now commercially available and being incorporated into infant formulas to narrow this gap (10, 11). While observational studies have provided valuable insights into the role of HMOs, a growing number of prospective human clinical trials are now focusing on the efficacy, safety, and health benefits of manufactured HMOs in various populations. These trials aim to assess the impact of HMOs in clinical settings, particularly in infant nutrition, gut microbiota composition, immune support, and disease prevention.

This review critically evaluates current evidence from clinical trials that specifically investigate the impact of manufactured HMOs on clinical applications across diverse populations ranging from pre-term infants to adults with or without medical conditions, excluding observational studies. It highlights key findings on the clinical applications of HMOs in infant nutrition, immune support, neurodevelopment, and disease prevention. Additionally, the review explores potential mechanisms of action identified in these clinical studies, addresses significant knowledge gaps, and outlines directions for future research.

## Methods

**Figure 1:**
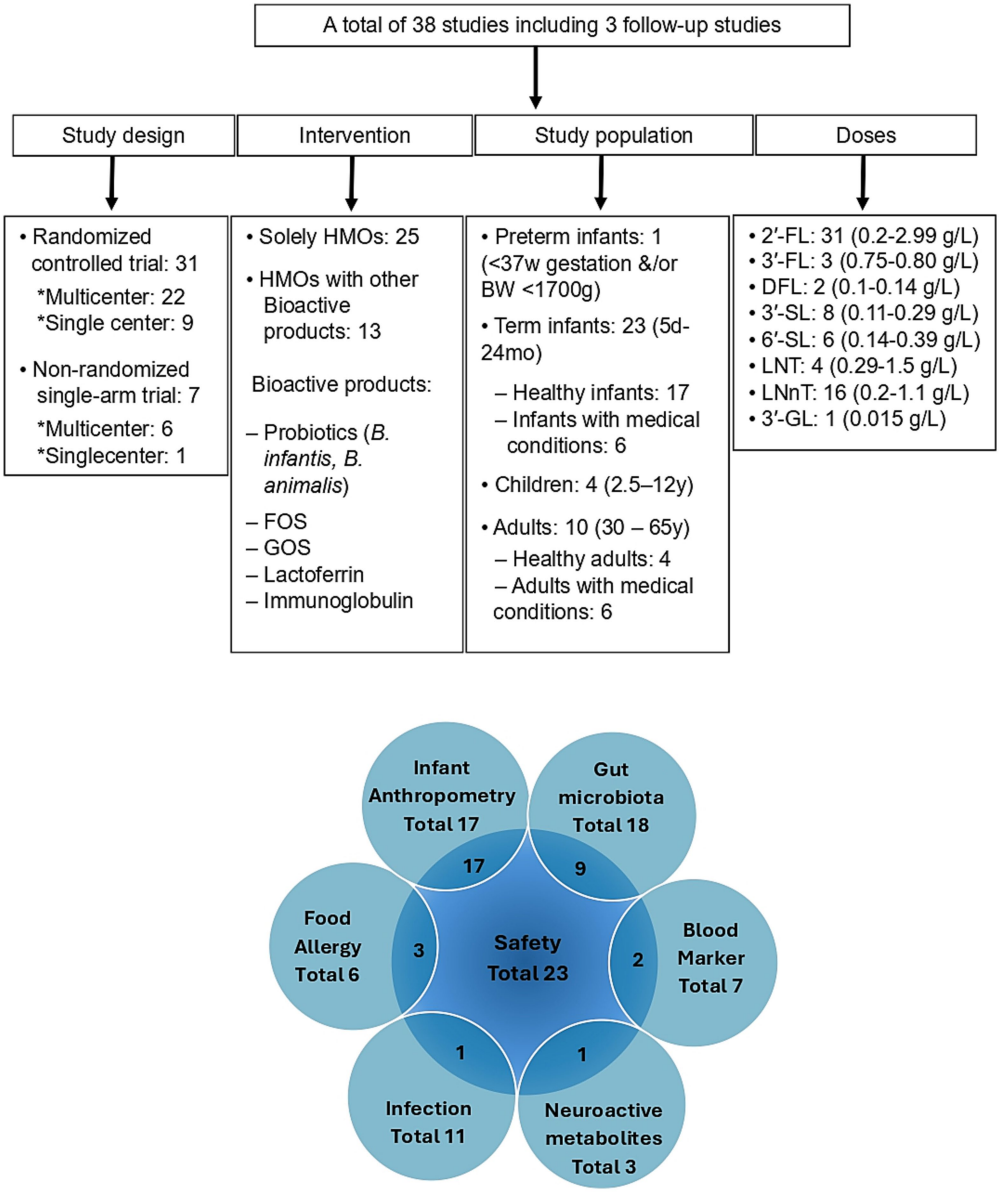
Summary of clinical intervention studies investigating the health effects of manufactured human milk oligosaccharides (HMOs), focusing on study population characteristics, administered dose ranges, and evaluated clinical endpoints. Children’s medical conditions are CMPA, food protein allergy/sensitivity, and feeding intolerance symptoms. Adult medical conditions are Ulcerative colitis, *Helicobacter pylori*, IBS, diabetes, obesity, and osteoarthritis. HMOs, Human milk oligosaccharides; 2’-FL, 2’-Fucosyllactose; 3’-FL, 3’-Fucosyllactose; DFL, Difucosyllactose; 3’-SL, 3’-Sialyllactose; 6’-SL, 6’-Sialyllactose; LNT, Lacto-N-tetraose; LNnT, Lacto-N-neotetraose; 3′-GL, 3′-Galactosyllactose; FOS, Fructo-oligosaccharides; GOS, Galacto-oligosaccharides; SCFAs, Short chain fatty acids; B, BW, Body weight; CMPA, Cow’s milk protein allergy; W, Week; mo, Month; Y, Year; d, Day.

A systematic literature search was performed in the PubMed, Medline, and Scopus electronic databases up to December 2024 without any restriction on the publication date. The search terms included “human milk oligosaccharide” AND “clinical trial”; “human milk oligosaccharide” AND “clinical intervention”; “human milk oligosaccharide” AND “infant formula.” Only original studies published in English were considered. Additionally, reference lists from primary articles and related reviews were examined to identify other studies for potential inclusion. The complete search strategy is shown in detail in Table 1. In brief, a total of 706 articles were initially retrieved. Following the elimination of duplicates (287) and the application of exclusion and inclusion criteria, a total of 38 articles were ultimately selected for this review. A summary of these studies including study design, HMO intervention, population range, doses, and outcomes, are presented in Figure 1, with further details outlined in Tables 2–4.

**Table 1:** Inclusion and exclusion criteria for the review of manufactured HMOs in clinical studies.

Inclusion criteria	Exclusion criteria
Clinical trials	Pre-clinical trials
Only published in the English language	Observational studies
Original article	Biosynthesis approach of HMOs
Manufactured HMOs as an oral intervention	Consumption of HMOs through breastfeeding
Supplementation of HMOs solely or combined with other food products	Exclusive supplementation with prebiotics other than HMOs
Individuals of all ages with or without medical conditions	Maternal interventions aimed at assessing outcomes in offspring

**Table 2:** Clinical studies investigating the effect of manufactured HMOs on healthy infants and children.

References	Study design	Study population/sample	Intervention group(s)	Comparison group	Duration of intervention	Outcome
Scheuchzer et al. (68)	RSBScCT	Healthy term infants (8–14 mo) (*n =* 76)	- FUF with HMO (100 mg/100 mL 2’-FL) - FUF + symbiotic (400 mg/100 mL GOS + *L. reuteri*)	- Control FUF without HMO or synbiotic	51 d	- No significant difference in FIA - FIA negatively associated with hemoglobin levels and fecal pH
Holst et al. (41)	RDBMcCT	Healthy infants (≤ 14 d) (*n =* 211, primarily 311 but excluded from different criteria)	- HMO (4.35 g 5-HMO mix/100 g formula+5.75 g/L including proteins, lipids, vits, and other nutrients) 5-HMOs (LNT, 2′FL, 3′-FL, 3′-SL&6′-SL) (*n =* 65)	- BFRG (*n =* 90) - IF grp 4.35 g Maltodextrin/100 g of formula with 5.75 g/L including proteins, lipids, vits, and other nutrients (*n =* 56)	6 mo	- ↑ Relative abundance of *Bifidobacteria* especially those that can produce aromatic lactic acids - ↓ Opportunistic pathogens to levels seen in BF infants
Jochum et al. (14)	NROMT	Healthy term infants (7 d to 2 mo) (*n =* 106)	- Exclusively infant FF (*n =* 46)- HMOs: 1 g/L 2′-FL + 0.5 g/L LNnT	- Breastfed (*n =* 38) - Mixed fed (both BF and FF) (*n =* 22)	8 wk	- Proven safe and having good tolerability - Promote age-consistent growth patterns - A small number of adverse events in FF infants
Alliet et al. (15)	RDBMcCT	Healthy term infants (<14 d at inclusion) (*n =* 289)	- 1 g/L 2′-FL + *L. reuteri* (*n =* 144)	- Control formula without OS + *L. reuteri* - BFRG (*n =* 60)	6 mo	- Proven safe and having good tolerability - Promote age-consistent growth patterns - Gut microbiota profile transitioned toward the pattern observed in BF infants
Bosheva et al. (44)	RDBMcCT	Healthy term infants (<21 d at inclusion) (*n =* 535)	- 1.5 g/L HMOs (0.87 g/L 2′-FL + 0.10 g/L DFL +0.29 g/L LNT + 0.11 g/L 3′SL + 0.14 g/L 6′-SL (*n =* 153) - 2.5 g/L HMOs (1.45 g/L 2′-FL + 0.14 g/L DFL + 0.48 g/L LNT + 0.18 g /L 3′-SL + 0.24 g/L 6′SL (*n =* 158)	- Control formula without OS (*n =* 155) - BFRG (*n =* 69)	6 mo	- Modulation of stool biomarkers - Gut microbiota profile transitioned toward the pattern observed in BF infants
Lasekan et al. (16)	RDBMcCT	Healthy term infants <14 d at inclusion (*n =* 363)	- 5.75 g/L HMOs (3 g/L 2′- FL + 0.8 g/L 3′-FL + 1.5 g/L LNT + 0.2 g/L 3′SL+ 0.3 g/L 6′SL) (*n =* 130)	- Control formula without OS (n = 129) - BFRG (*n =* 104)	4 mo	- Proven safe and having good tolerability - Promote age-consistent growth patterns - Decreased frequency of healthcare visits - Fecal characteristics resembled those observed in BF Infants
Martin et al. (63)	RDBMcCT	Healthy term infants (<14 d) (*n =* 80)	- 1.5 g/L HMOs (1 g/L 2′-FL + 0.5 g/L LNnT) (*n =* 38)	- Control (1.5 g/L Lactose) (*n =* 42)	4 mo	-HMOs influenced three key molecular pathways: Gamma-glutamylation and N-acetylation of amino acids, and reduction of inflammatory signaling lipids -↑ Abundance of beneficial bacteria (*Bifidobacterium* and *Bacteroides*)
Wallingford et al. (17)	RDBMcCT	Healthy term infants (<28 d at inclusion) (*n =* 221)	- 1 g/L 2′-FL (*n =* 66)	- Control formula + GOS + FOS (dose not specified) (*n =* 66) - BFRG (*n =* 89)	16 wk	- Proven safe and promote age-consistent growth patterns - ↑ Microbial functional capacity for fucosylated HMO utilization - Gut microbiota profile transitioned toward the pattern observed in BF infants
Dogra et al. (45)	Follow-up study of (18)				6 mo follow-up without intervention	- Impact on fecal biomarkers - Gut microbiota profile transitioned toward the pattern observed in BF infants
Iribarren et al. (33)	Follow-up analysis of (36)				4-wk follow-up (no HMO intervention)	- ↑ Fecal and mucosal *Bifidobacterium* - Modulate fecal and plasma biomarkers
Parschat et al. (19)	RDBMcCT	Healthy term infants (<14 d at inclusion) (*n =* 341)	- 5.75 g/L HMOs (2.99 g/L 2′-FL + 0.75 g/L 3′-FL + 1.5 g/L LNT + 0.23 g/L 3′-SL + 0.28 g/L 6′-SL (*n =* 113)	- Control formula without OS (*n =* 112) - BFRG (*n =* 116)	16 wk. intervention + 8-wk voluntary follow-up	- Proven safe and having good tolerability - Promote age-consistent growth patterns - Fecal characteristics resembled those observed in BF infants
Berger et al. (69)	Follow-up analysis of (18)		- Subpopulation from 1 g/L 2′-FL + 0.5 g/L LNnT (*n =* 58)	- Subpopulation control formula without OS (*n =* 64) - Subpopulation BFRG (*n =* 35)	12 mo	- Gut microbiota profile transitioned toward the pattern observed in BF infants than in the control group without OS - Higher levels of *Bifidobacteriaceae* resemblance to BF infants associated with reduced antibiotic use
Roman et al. (20)	NROMcT	Healthy term infants (7 d to 2 mo at inclusion) (*n =* 207)	- 1 g/L 2′-FL + 0.5 g/L LNnT (Partially hydrolyzed formula) (*n =* 66)	- Mixed group (HMO formula + BF) - BF reference group (*n =* 45)	8 wk	- Proven safe and having good tolerability - Promote age-consistent growth patterns
Vandenplas et al. (21)	RDBMcCT	Healthy term infants (<14 d at inclusion) (*n =* 276)	- 1 g/L 2′-FL + 7.2 g/L GOS + 0.8 g/L FOS, including 0.015 g/L 3′- GL (Partially hydrolyzed formula) (*n =* 108)	- Control formula without 2′-FL and 3′-GL, with 7.2 g/L GOS + 0.8 g/L FOS (*n =* 107) - BF reference group (*n =* 61)	17 wk	- Proven safe and having good tolerability - Promote age-consistent growth patterns
Storm et al. (22)	RDBMcCT	Healthy term infants (14 ± 5 d at inclusion) (*n =* 79)	- 0.25 g/L 2′-FL + *Bifidobacterium animalis* sp. *lactis* (partially hydrolyzed formula) (*n =* 39)	- Control formula without OS + *Bifidobacterium animalis* sp. *lactis* (*n =* 40)	6 wk	- Proven safe and having good tolerability - Promote age-consistent growth patterns - Progressive decline in reported infection case
Puccio et al. (18)	RDBMcCT + Follow-up study	Healthy term infants (<14 d at inclusion) (*n =* 175)	- 1 g/L 2′-FL + 0.5 g/L LNnT (*n =* 88)	- Control formula without OS (*n =* 87) - BFRG (*n =* 38)	6 mo (HMO intervention) + follow-up up to 12 mo	- Proven safe and having good tolerability - Promote age-consistent growth patterns - ↓ LRTIs linked to ↓ acetate and ↑ *Bifidobacteria* Proportions - ↓Prevalence of infantile colic at 4 months - ↓ Bronchitis and ↓ pharmacological intervention during the first year of life
Goehring et al. (43)	Follow-up analysis of (23)	Subset of study (23) Healthy term infants (<5 d at inclusion) (*n =* 315)	- Subpopulation from: 0.2 g/L 2′-FL + 2.2 g/L GOS (*n =* 76) - Subpopulation from 1 g/L 2′-FL + 1.4 g/L GOS (*n =* 78)	- Subpopulation from control formula: 2.4 g/L GOS (*n =* 75) - Subpopulation from BFRG (*n =* 86)	4 mo follow up	- Comparable cytokine profiles and peripheral blood mononuclear cell activation to those of BF groups
Kajzer et al. (24)	RDBMcCT	Healthy term infants (<8 d at inclusion) (*n =* 119)	- 0.2 g/L 2′-FL + 2 g/L scFOS (*n =* 46)	- Control formula without OS (*n =* 42) - BFRG (*n =* 43)	35 d	- Having good tolerability - Promote age-consistent growth patterns
Marriage et al. (23)	RDBMcCT	Healthy term infants (<5 d at inclusion) (*n =* 420)	- 0.2 g/L 2′-FL + 2.2 g/L GOS (*n =* 104) - 1 g/L 2′-FL + 1.4 g/L GOS (*n =* 109)	- Control formula: 2.4 g/L GOS (*n =* 101) - BFRG (*n =* 106)	17 wk	- Proven safe and having good tolerability - Promote age-consistent growth patterns - Comparable relative absorption and excretion of 2′-FL to those of BF groups at the life days 42 and 119
Prieto (13)	RDBMcCT	Healthy term infants aged 6–24 mo (*n =* 228)	- 0.2 g/L LNnT (*n =* 115)	- Control formula without OS (*n =* 113)	16 wk	- Proven safe and having good tolerability - No impact on *Streptococcus pneumoniae* colonization of the oropharynx
Bajic et al. (28)	RCScT	Healthy children (6 y) (*n =* 6) and adults (average 30 y) (*n =* 6)	- 3 g/d 2′-FL, 1.8 g/d LNnT, 0.5 g/d 3′-SL, 0.8 g/d 6′-SL	- Control group: No-substrate	0, 6, 24, and 48 h	- ↑ Gut–brain-axis-related metabolites (SCFAs, exhibiting greater effects with increased dosage)

2’-FL, 2’-Fucosyllactose; 3′-GL, 3′-Galactosyllactose; 3’-SL, 3’-Sialyllactose; 6’-SL, 6’-Sialyllactose; BF, Breast-fed; BFRG, Breastfed reference group; d, Day; DFL, Difucosyllactose; FF, Formula-fed; FOS, Fructo-oligosaccharides; FUF, Follow-up-formula; g/d, Gram per day; g/L, Gram per litre; g, Gram; GOS, Galacto-oligosaccharides; HMO, Human Milk Oligosaccharides; IF, Infant formula; L, Limosilactobacillus; LNnT, Lacto-N-neotetraose; LNT, Lacto-N-tetraose; mg, Milligram; mL, Milliliter; mo, Month; OS, Oligosaccharides; SCFAs, Short chain fatty acids; scFOS, Short-chain fructo-oligosaccharides; sp, Species; Vits, Vitamins; wk, Week; y, Year. In study design, R, Randomized; NR, Non-randomized; C, Controlled; T, Trial, Sc, Single-center; Mc, Multi-center; SB, Single-blind; DB, Double-blind; TB, Triple-blind, P, Pilot; DBPCFC, Double-blind, placebo-controlled food challenges; OFC, Open food challenge.

**Table 3:** Clinical studies investigating the effect of manufactured HMOs on pre-term infants, full-term infants, and children with medical conditions.

References	Study design	Study population/sample	Intervention group(s)	Comparison group	Duration of intervention	Outcome
Hascoet et al. (30)	RDBMcCT	Pre-term infants with very low birth weight (<1700 g) (*n =* 86)	- 0.34 g/kgbw/d 2′FL + 0.034 g/kg bw/d LNnT (*n =* 43)	- Placebo (0.140 g/kgbw/d Glucose, *n =* 43)	Average 45 d (lasted until neonatal unit discharge)	- Proven safe and having good tolerability - Greater average head circumference growth by day 21 before complete enteric feeding - Higher length-for-age z-score
Ramirez-Farias et al. (31)	NRMcT	Infants (< 90 d) experiencing persistent feeding intolerance symptoms or symptoms of suspected food protein allergy (*n =* 33)	- 0.2 g/L of 2′-FL, DHA, ARA, and nucleotides	- No control group	28 d	- Proven safe and having good tolerability - Promote age-consistent growth patterns - Mitigated symptoms of allergy and food intolerance
Boulange et al. (42)	RDBMcCT	Non-BF infants with CMPA (mean age 3.2 mo) (*n =* 194)	- 1 g/L 2′-FL, 0.5 g/L LNnT	- HMO-free Control (*n =* 72)	12 mo	- Partially improved dysbiosis observed in infants with CMPA - Gut microbiota profile transitioned toward the pattern observed in BF infants - Reduce respiratory and gastrointestinal infections
Gold et al. (25)	NROMcT	Term infants (1–8 mo) diagnosed with moderate-to-severe CMPA (*n =* 32)	- 1 g/L 2′-FL + 0.5 g/L LNnT (Amino acid-based formula) (*n =* 32)	- No control group	4 mo + voluntarily up to 12 mo of age	- Proven safe and having good tolerability - Promote age-consistent growth patterns - Gut microbiota profile and fecal characteristics transitioned toward the pattern observed in BF infants - ↑ SCFA concentrations in feces - Improved allergy symptoms
Vandenplas et al. (26)	RDBMcCT	Term infants (0–6 mo) diagnosed with CMPA (*n =* 194)	- 1 g/L 2′-FL + 0.5 g/ L LNnT (Extensively hydrolyzed formula, *n =* 94)	- Control formula without OS (*n =* 96)	4 mo + follow-up to 12 mo	- Proven safe and having good tolerability - Promote age-consistent growth patterns - No impact on allergy - Lower incidence of URTIs and decreased risk of otitis media - ↓ Use of antipyretics
Ramirez-Farias et al. (27)	NRMcT	Infants < 60 d with suspected food protein allergy or sensitivity (*n =* 48)	- 0.2 g/L 2′-FL (Extensively hydrolyzed formula, *n =* 48)	- No control group	60 d	- Proven safe and having good tolerability - Promote age-consistent growth patterns - Mitigated allergy
Nowak-Wegrzyn et al. (70)	DBPCFC and OFC	Infants and children (2 mo and 4 y) with diagnosed CMPA (*n =* 67)	- 1 g/L 2′-FL + 0.5 g/L LNnT (Extensively hydrolyzed formula, *n =* 36 in DBPCFC, *n =* 62 in OFC)	- Control formula without OS (*n =* 31, only in DBPCFC)	1 wk	- Proven safe and having good tolerability - Demonstrated hypo-allergenicity
Fonvig et al. (34)	RDBScCT	Overweight/obese children (6–12 y, *n =* 75)	- 4.5 g/d 2′-FL (*n =* 25) - 4.5 g/d HMO (3.6 g/d 2′-FL + 0.9 g/d LNnT *n =* 25)	- Placebo (4.5 g/d Glucose, *n =* 25)	8 wk	- Proven safe and having good tolerability - Impact on gut microbiota profile - No impact on fecal and blood markers

2’-FL, 2’-Fucosyllactose; ARA, Arachidonic acid; BF, Breast-fed; CMPA, Cow’s milk protein allergy; d, Day; DHA, Docosahexaenoic acid; g/d, Gram per day; g/kgbw/d, Gram per kg body weight per day; g/L, Gram per litre; g, Gram; HMO, Human Milk Oligosaccharides; LNnT, Lacto-N-neotetraose; mg, Milligram; mo, Month; OS, Oligosaccharides; SCFAs, Short chain fatty acids; URTIs, Upper respiratory tract infections; wk, Week; y, Year. In study design, R, Randomized; NR, Non-randomized; C, Controlled; T, Trial; Sc, Single-center; Mc, Multi-center; SB, Single-blind; DB, Double-blind; P, Pilot; DBPCFC, Double-blind, placebo-controlled food challenges; OFC, Open food challenge.

**Table 4:** Clinical studies investigating the effect of manufactured HMOs on adults with or without medical conditions.

References	Study design	Study population/sample	Intervention group(s)	Comparison group	Duration of intervention	Outcome
Kim et al. (12)	RTBMcCT	Healthy adults (≥20 y) (*n =* 60)	- 3 g/d 6′-SL (*n =* 30)	- Placebo (3 g/dMaltodextrin, *n =* 30)	12 wk	- Proven safe and having good tolerability - No effect on blood markers
Elison et al. (35)	RDBScCT	Healthy adults (18–60 y) (*n =* 100)	- 5 g/d 2′-FL (*n =* 10) - 10 g/d 2′-FL (*n =* 10) - 20 g/d 2′-FL (*n =* 10) - 5 g/d LNnT (*n =* 10) - 10 g/d LNnT (*n =* 10) - 20 g/d LNnT (*n =* 10) - 5 g/d HMO (3.34 g/d 2′-FL + 1.66 g/d LNnT, *n =* 10) - 10 g/d HMO (6.68 g/d 2′ -FL + 3.32 g/d LNnT *n =* 10) - 20 g/d HMO (13.36 g/d 2′-FL + 6.64 g/d LNnT, *n =* 10)	- Placebo (2 g/d glucose) (*n =* 10)	2 wk	- Demonstrated safety and well-tolerability at a daily intake of 20 g of 2′-FL and LNnT - Gastrointestinal discomfort in certain higher-dose intervention groups - ↑ Abundance of *Actinobacteria* and *Bifidobacterium* accompanied by ↓ abundance of *Firmicutes* and *Proteobacteria* - No impact on fecal and blood markers
Canfora et al. (53)	RDBScT (crossover)	Lean men (*n =* 10) men with prediabetes and overweight/obesity (*n =* 12), aged 30–65 y	- 12 g 2′-FL + 5.43 g maltodextrin - 12 g 2′-FL + 9.39 g resistant starch	Placebo (11.43 g maltodextrin)	1 d (wash out period 14 d between interventions)	- ↑ SCFA (butyrate and acetate) which lowered plasma FFA
Ko et al. (39)	RDBScCT	Overweight and sedentary adults (25–51 y, BMI: 31.6 ± 6.6 kg/m^2^) (*n =* 41)	- 3 g/d momstamin 2’-FL + Exercise (10,000 steps/day, 5 days/week) + Energy-reduced diet (300 kcal/d)	- Placebo (3 g/day of maltodextrin + same exercise and diet intervention) (*n =* 20)	12 wk. - baseline, 6-wk and 12-wk follow-up	- No significant difference in weight loss between groups - 2′-FL group had a greater fat loss, less muscle loss, and higher fat oxidation - No significant differences in waist/hip circumference - Improved aerobic capacity, IL-4, platelet aggregation, and functional capacity perception
Park et al. (65)	RSBScPT	Adults (≥40 y) with knee OA	- 200 mg/d 3′SL (*n =* 20) 600 mg/d 3′SL (*n =* 20)	- Placebo (1tablet micro-crystalline) cellulose (*n =* 20)	12 wk	- Pain intensity scores significantly reduced at 12 wk. in both 3′-SL groups - KWOMAC physical function scores improved at 12wk
Iribarren et al. (36)	RDBScCT	Adults with IBS (18–75 y) (*n =* 61)	- 5 g/d HMO (4 g/d 2′ FL + 1 g/d LNnT) (*n =* 20) - 10 g/d HMO (8 g/d 2′- FL + 2 g/d LNnT) (*n =* 20)	- Placebo (5 g/d Glucose) (*n =* 21)	4 wk.	- Having good tolerability - No exacerbation of IBS - No impact on gut microbiota profile
Ryan et al. (40)	NROMcPT	Adults with IBS, ulcerative colitis, Crohn’s disease, or celiac disease (21–75 y, *n =* 20)	- 4 g/d 2′-FL (*n =* 20)	- No control group	6 wk	- Improved IBS symptoms - ↑ Abundance of *Bifidobacteria* and other beneficial bacteria *Clostridium* cluster XIVa and *Roseburia* sp. (butyrate producers) - ↑ SCFA concentrations including butyrate in feces
Palsson et al. (32)	NROMcT	Adults (average 44 y) with IBS (*n =* 317)	- 4 g/d 2′-FL + 1 g/d LNnT (*n =* 317)	- No control group	12 wk	- Proven safe and having good tolerability - Relieved IBS-related discomfort - Promotes normal bowel patterns in IBS by balancing stool consistency
Parente et al. (37)	RDBScCT	Adults (44–62 y) with diagnosed *H. pylori* infection (*n =* 65)	- 10 g/d 3′-SL (*n =* 17) - 20 g/d 3′-SL (*n =* 22)	- Placebo (not Specified, *n =* 21)	4 wk	- No impact on *H. pylori* infection - Having good tolerability
Opekun et al. (71)	OT	Adults (33–49 y) with diagnosed *H. pylori* infection (*n =* 6)	- 10 g/d 3′-SL (*n =* 6)	- No control group	1 d	- Having good tolerability - No impact on *H. pylori* infection - No impact on blood markers

2’-FL, 2’-Fucosyllactose; 3’-SL, 3’-Sialyllactose; 6’-SL, 6’-Sialyllactose; BMI, Body mass index; d, Day; FFA, Free fatty acid; FIA, Fractional iron absorption; g/d, Gram per day; g, Gram; H, Helicobacter; HMO, Human Milk Oligosaccharides; IBS, Irritable bowel syndrome; kcal/d, Kilocalories per day; kg/m^2^, Kilograms per square meter; KWOMAC, Korean Western Ontario and McMaster Universities osteoarthritis index; LNnT, Lacto-N-neotetraose; mg, Milligram; mo, Month; OA, Osteoarthritis; SCFAs, Short chain fatty acids; sp, Species; VAS, Visual analog scale; wk, Week; y, Year. In study design, R, Randomized; NR, Non-randomized; C, Controlled; T, Trial, Sc, Single-center; Mc, Multi-center; SB, Single-blind; DB, Double-blind; TB, Triple-blind, O, Open-label; P, Pilot; DBPCFC, Double-blind, placebo-controlled food challenges.

### Safety assessment of manufactured HMO intervention

The primary safety endpoints assessed in these clinical trials are as follows:

- Gastrointestinal tolerance: incidence of diarrhea, constipation, bloating, gas, or vomiting.
- Allergic reactions: monitoring hypersensitivity, rashes, or anaphylaxis.
- Metabolic effects: blood glucose levels, lipid profiles, and metabolic markers.
- Immune response: biomarkers of inflammation and immune activation.
- Microbiota composition: changes in gut microbial diversity and balance.

Most of the reviewed studies do not explicitly report structural verification of HMOs (e.g., 2’-FL and 6’-SL) to ensure accurate manufacturing. Additionally, detailed source verification, such as confirming the absence of microbial contaminants and DNA residues for microbial fermentation-derived HMOs or chemical contaminants for enzymatically synthesized HMOs is often missing. Addressing these points is critical for ensuring product safety and consistency. However, a few studies have incorporated these quality control measures. For instance, Kim et al. (12) verified the identity of enzymatically synthesized 6′-SL by confirming a purity of 98.8% and the presence of Sia content. The source was validated as non-pathogenic *E. coli*, with assurances of safety through the absence of microbial contaminants (12). Similarly, Prieto (13) described rigorous production and purification methods for LNnT, employing mass spectrometry and chromatography to eliminate contaminants. These studies highlight the importance of integrating robust quality control measures into HMO research.

The safety of HMOs has been evaluated across diverse populations, including pre-term and term infants, children, and adults, with or without medical conditions or food allergies. The safety of HMOs has primarily focused on three key aspects: growth measures, adverse events (AEs), and gastrointestinal tolerance. Growth measures were assessed using anthropometric parameters to evaluate the nutritional adequacy of HMOs (14–29). These measures provide insights into whether HMO supplementation supports normal growth and development, particularly in infants. AEs were recorded through validated questionnaires completed by parents or caregivers and medical confirmation provided by physicians (15–17, 19, 21, 25, 29–32). These AEs included infections, such as gastrointestinal, respiratory tract, and ear infections, allergic reactions like skin rashes and hypersensitivity responses, and general medical conditions such as pyrexia and other mild to moderate illnesses. AEs were classified based on severity (mild, moderate, or severe). The details of growth measures and AEs are elaborated separately under the sections “Infant Anthropometric Parameters” and “Infection” in this article.

Gastrointestinal tolerance was assessed by using standardized tools, including the Gastrointestinal Symptom Rating Scale (GSRS) (33, 34), Gastrointestinal Symptom Questionnaire (GISQ) (22), and the Bristol Stool Form Scale (BSFS) (34). Monitored symptoms included diarrhea, vomiting, gastric residual volumes, flatulence, and stool consistency and frequency. Dose–response studies established safe dosage levels for pre-term infants to term infants irrespective of their medical conditions. For pre-term infants, a daily dose of 0.35 g/kg body weight of 2’-FL and LNnT in a 10:1 ratio was established as safe (30). However, in healthy term infants, the combination of 1 g/L 2’-FL and 0.5 g/L LNnT demonstrated safety and tolerability (21–23, 27). In HMO blends, a combination of five HMOs, including neutral, sialylated, and fucosylated oligosaccharides, was found to be safe at 5.75 g/L (19). Furthermore, in children with or without medical conditions such as cow’s milk protein allergy (CMPA) or other food allergies, a 4.5 g/L dose of 2’-FL, in a 4:1 ratio with LNnT, demonstrated excellent tolerability (25, 26, 29, 34).

In healthy adults, higher doses of HMOs have been investigated, revealing nuanced gastrointestinal effects. A daily intake of 20 g of 2’-FL led to increased abdominal rumbling, while the same dose of LNnT resulted in harder stools. However, a mixture of 2’-FL and LNnT showed better tolerability than the individual oligosaccharides at these high doses (35). In patients with irritable bowel syndrome (IBS), daily intake of a 4:1 mix of 2′-FL and LNnT at doses of 5 g or 10 g over 4 weeks significantly increased *Bifidobacterium* spp. abundance, modulated fecal and plasma metabolite profiles, and improved IBS symptoms—all without inducing adverse immune or mucosal responses—supporting a favorable safety profile (33, 36). Complementing this, a more extensive 12-week open-label study involving 317 IBS patients confirmed that daily supplementation with 5 g of the same 4:1 mix of 2′FL and LNnT improved bowel function, reduced IBS symptom severity, and enhanced quality of life, with only mild gastrointestinal discomforts reported as side effects (32). Additionally, in a placebo-controlled trial among dyspeptic adults, daily administration of 3-′SL at 10 g or 20 g was well tolerated. However, it did not eradicate *Helicobacter pylori* infection, indicating that even high doses of isolated HMOs can be safely consumed in humans (37). Collectively, these findings support the safety and tolerability of manufactured HMOs across diverse populations and clinical contexts while also underscoring the need for standardized quality control to ensure consistent formulation. Consequently, the optimal HMO dose and composition may need to be tailored to the target population and the intended physiological or clinical outcome.

### Functional impacts of HMOs

#### Anthropometric parameters

The nutritional adequacy of HMOs has been widely evaluated through their impact on growth and development, as measured by weight, length, head circumference, waist/hip circumference, and body mass index (BMI). In pre-term infants, HMO-enriched formulas containing 2′-FL and LNnT have demonstrated a faster transition to full enteral feeding compared to placebo, with no adverse effects reported. Growth outcomes in these infants were comparable to standard feeding practices, with increased length-for-age z-scores, indicating a positive role in postnatal growth and development (30). In healthy-term infants, studies adhering to World Health Organization (WHO) growth standards have compared weight-for-age and length-for-age z-scores, across HMO-enriched formulas, breastfeeding, and standard formula groups (14–29). The results demonstrated that infants consuming HMO-enriched formula exhibited non-inferior growth compared to the breastfed reference group. Furthermore, growth outcomes in the HMO-formula group were superior to those observed in infants fed standard formula. Moreover, in infants with dietary sensitivities—such as persistent feeding intolerance or suspected food protein allergies—a 28-day intervention with hydrolyzed rice infant formula (HRF) enriched with 2′-FL resulted in statistically significant improvements in feeding tolerance (31). Head circumference, a critical neurodevelopmental marker, has also been examined in several clinical studies (14, 17, 19, 31). Parshat et al. (19) demonstrated that an infant formula containing a mix of five HMOs (5HMO-Mix), including sialylated HMOs, supported head circumference growth within the normal range, showing non-inferiority compared to breastfed infants. While this finding does not establish a direct link, it suggests a potential role of sialylated HMOs, such as 3′-SL and 6′-SL, in contributing to early neurodevelopment, possibly through the provision of sialic acid (Sia), a key nutrient for brain growth (38). Additionally, a recent placebo-controlled study investigated the effects of 2′-FL supplementation (3 g/day) combined with a calorie-reduced diet and structured exercise regimen on body composition over 12 weeks in overweight adults (39). While the 2′-FL group showed improvements such as fat mass reduction, better fat oxidation, and loss of lean mass compared to baseline, these effects were not significantly different from those in the placebo group when adjusted for group-by-time interactions. Moreover, an increase in work-related physical activity was observed, which may have contributed to changes in body composition independent of the intervention. Although the results suggest a potential role for 2′-FL in modulating metabolic health markers, these findings should be interpreted cautiously due to the study’s modest sample size, non-placebo-adjusted primary outcomes, and variability in lifestyle behaviors. However, clinical trials assessing the direct effects of HMOs, particularly 2′-FL, on infant body composition and fat regulation remain limited. Furthermore, evidence gaps remain regarding the effects of specific HMOs in low-resource settings, where challenges such as postnatal linear growth faltering, undernutrition, and stunting persist. Clinical studies investigating the effects of manufactured HMOs on healthy infants and children, as well as those with medical conditions, are shown in Tables 2, 3.

#### Infections

As bioactive constituents of human milk, HMOs provide both direct and indirect protection against infections caused by pathogenic microorganisms. Puccio et al. (18) reported that healthy infants consuming a formula with 1.0 g/L 2′-FL and 0.5 g/L LNnT for 6 months experienced significantly fewer parental reports of bronchitis and lower respiratory tract infections (LRTIs) (18). A marked reduction in the use of antibiotics and antipyretics further suggests potential respiratory health benefits (18). Similarly, Vandenplas et al. (26) demonstrated that infants with CMPA receiving an extensively hydrolyzed formula (EHF) supplemented with these HMOs for 4 months showed a statistically significant reduction in the frequency of upper respiratory tract infections (URTIs) and a lower incidence of ear infections at 12 months. Additionally, the relative risk of LRTIs and gastrointestinal infections was reduced by 30–40%, underscoring the broad protective effects of HMO supplementation in vulnerable populations.

Leung et al. (29), however, observed that 2′-FL supplementations in healthy Chinese children aged 1–2.5 years over 6 months extended the duration of URTIs in some cases, suggesting that HMO effects may vary depending on demographic or health context (29). In contrast, Prieto (13) reported no significant effect of LNnT supplementation on oropharyngeal colonization with *Streptococcus pneumoniae* in children aged 6–24 months in Chile following a 16-week intervention. Beyond respiratory health, HMOs have been investigated for their potential benefits in gastrointestinal disorders (32, 40). Palsson et al. (32) found that a 5-g intervention with HMOs (2′-FL and LNnT in a 4:1 ratio) over 12 weeks significantly improved bowel movement consistency in patients with IBS, particularly within the first 4 weeks. Similarly, Ryan et al. (40) observed improvements in both intestinal and extra-intestinal symptoms, alongside bifidogenic and butyrogenic effects, in IBS and ulcerative colitis patients consuming 2′-FL-enriched nutritional formulas.

While HMOs appear beneficial across multiple domains, their role in urinary tract infections (UTIs) remains unclear. Irribaren et al. (33) detected 2′-FL in urine, but no clinical findings have confirmed its role in preventing UTIs by inhibiting bacterial adhesion to the urothelial lining. Collectively, these findings have demonstrated that supplementation with specific HMOs can reduce the incidence of infections in infants and adults. However, mechanistic insights remain limited or inconsistent across trials. Proposed pathways include the direct inhibition of pathogen adhesion to epithelial surfaces by acting as decoy receptors (35, 41) as well as the modulation of gut microbiota composition (15, 42)—particularly the enrichment of *Bifidobacterium* spp. and strengthening of the intestinal epithelial barrier. Additionally, HMOs are thought to influence immune responses through the regulation of cytokine production and enhancement of mucosal immunity (34, 43). Despite these promising leads, most clinical trials have not concurrently measured molecular markers of these mechanisms, limiting the ability to establish a causal relationship. Therefore, future research should integrate mechanistic endpoints with clinical outcomes to better elucidate how HMOs exert their protective effects against infections.

#### Gut microbiota and benefits

HMOs significantly influence the composition, function, and diversity of the gut microbiota, particularly during early life. Microbiome diversity is assessed by evaluating *α*-diversity (richness and Shannon index) and *β*-diversity (weighted UniFrac distances). α-diversity indicates the overall diversity within the gut microbiome of individual infants, while β-diversity highlights differences in microbiome composition between groups. Supplementation of infant formula with five HMOs [2’-FL, 3’-FL, Lacto-N-tetraose (LNT), 3’-SL, and 6’-SL] at a total natural concentration of 5.75 g/L induced distinct shifts in β-diversity, aligning the microbial composition more closely with that of breastfed infants (41). Similarly, Fonvig et al. (34) observed a significant increase in *α*-diversity after 8 weeks of supplementation with a mix of 2′-FL and LNT in infants. In contrast, no significant changes were observed in the placebo group or those receiving only 2’-FL at a dose of 4.5 g/day (34). While lower doses of 2’-FL or LNnT alone did not influence fecal alpha diversity, higher doses (10 g of 2’-FL /LNnT) modulated fecal microbial *β* diversity profiles (33).

This clinical evidence supports the role of HMOs as prebiotics, preferentially stimulating the growth and activity of beneficial bacteria such as *Bifidobacterium, Bacteroides*, and *helicon* strains in infants and children (15, 17, 19, 25, 42, 44, 45). A diverse and abundant bifidobacterial community during early life is associated with positive extended health outcomes (46). In contrast, reduced abundance and diversity of *Bifidobacteria* have been linked to medical conditions such as allergies (47), dermatitis (48, 49), and pediatric obesity (50). Fonvig et al. (34) demonstrated that infant formula supplemented with 2′-FL and LNnT significantly increased *Bifidobacteria* abundance in overweight children after 4 and 8 weeks. In addition, an amino acid-based formula (AAF) supplemented with these two HMOs significantly enriched HMO-utilizing *Bifidobacteria* and reduced the abundance of fecal *Proteobacteria* in infants with CMPA. These findings suggest that the HMO-supplemented formula may help to correct gut microbial dysbiosis in CMPA infants (25).

Beyond promoting beneficial microbes, HMOs act as decoy receptors, preventing pathogenic bacteria, such as *Escherichia coli and Clostridium difficile,* from adhering to the intestinal epithelium (35, 41). Elison et al. (35) further demonstrated that HMO supplementation in adults led to substantial increases in *Actinobacteria* and *Bifidobacterium* while reducing Firmicutes and Proteobacteria, reinforcing their bifidogenic effects.

#### Blood and fecal markers

*Blood marker:* HMOs play a pivotal role in modulating immune function and gut health. Plasma cytokine concentrations and the composition of major lymphocyte subsets within peripheral blood mononuclear cells (PBMCs) serve as key biomarkers of immune functions. Goehring et al. (51) provided the first direct evidence that 2′-fucosyllactose (2′-FL) is present in the systemic circulation of both breastfed infants and those fed formula supplemented with 2′-FL, with plasma concentrations ranging from 0.1 to 4.0 mg/L, as measured using a validated liquid chromatography–mass spectrometry (LC–MS) method (51). Although human milk oligosaccharides (HMOs) are generally considered non-digestible and are thought to reach the colon intact, these findings suggest that a fraction of HMOs—particularly smaller, neutrally charged structures like 2′-FL—may be absorbed in the small intestine and transported into the bloodstream. The authors propose that this absorption may occur via paracellular pathways, such as through tight junctions between intestinal epithelial cells (51). Similarly, Irribaren et al. (33) identified the presence of 2’-FL in plasma, highlighting its systemic absorption and implying potential roles in immune modulation. However, further studies are needed to elucidate the metabolic fate of 2′-FL and confirm these findings. The presence of 2′-FL in circulation has been associated with reduced plasma concentrations of inflammatory cytokines in both breastfed infants and those receiving 2′-FL-enriched formula. For example, Goehring et al. (43) reported that infants fed 2’-FL-enriched formula exhibited significantly lower plasma levels of IL-1*α*, IL-1β, IL-6, and TNF-α compared to control groups, reflecting innate cytokine profiles more closely resembling those of breastfed infants. Notably, a higher concentration of 2′-FL (1 g/L) did not demonstrate greater efficacy than the lower dose (0.2 g/L) in altering cytokine profiles. The potential mechanisms underlying these anti-inflammatory effects induced by 2′-FL may involve *Bifidobacteria,* which utilize HMOs as their sole carbon source and suppress inflammatory gene expression in colonic epithelial cells (52). Furthermore, 2’-FL supplementation has been shown to narrow the differences in adaptive immune parameters, including the proportions of total T lymphocytes (helper and cytotoxic T cells) and the percentages of apoptotic cells, particularly CD8 + T cells, among infants who are breastfed and those who are formula-fed (43). This modulation of apoptosis—a crucial process for cellular homeostasis and immune regulation—emphasizes the multifaceted role of 2’-FL in supporting immune development. However, not all studies demonstrate consistent effects. Fonvig et al. (34) observed no significant changes in blood markers related to inflammation, such as IL-6, IL-8, IL-10, TNF-*α*, and C-reactive protein in obese children supplemented with 2’-FL and LNnT over 8 weeks. This variability underscores the influence of demographic and health factors on immune responses to HMOs.

Regarding nutritional biomarkers, Kim et al. (12) reported no significant changes in blood glucose, cholesterol, or triglycerides in healthy adults supplemented with 6’-SL for 12 weeks, confirming the metabolic safety of HMOs at clinical doses (3 gm/day). Canfora et al. (53) reported that 2′-FL supplementations significantly increased systemic butyrate levels in lean individuals and those with prediabetes or obesity. This elevation in butyrate was linked to improved metabolic health markers, including reductions in plasma-free fatty acids and inflammation. Interestingly, acetate levels increased only in lean men, highlighting a phenotype-specific response to supplementation. Consistent with these observations, Bajic et al. (28) reported dose-dependent increases in acetate, propionate, and butyrate following HMO supplementation, starting from estimated doses of 0.3–0.5 g/day. Their findings highlighted distinct effects of individual HMOs: 6′-SL had the strongest impact on propionate, 2’-FL and 3′-SL significantly elevated acetate, and LNnT notably increased butyrate, a key molecule associated with neuroactive pathways due to its capacity to traverse the blood–brain barrier and influence neurotransmitter synthesis (54).

*Fecal marker:* Fecal metabolites serve as indicators of gut microbial function and play a critical role in host health. Iribarren et al. (55) observed distinct modulation of metabolite profiles, including changes in asparagine, an amino acid associated with intestinal barrier integrity, and tryptophan, another essential amino acid linked to the pathogenesis of IBS (56). Tryptophan also serves as a precursor to serotonin, which is vital for mood regulation, cognitive function, and sleep (57). These changes were more pronounced in the intervention group receiving 10 g 2′-FL/LNnT than those receiving 5 g 2′-FL/LNnT or a placebo.

Furthermore, the untargeted metabolomic analysis demonstrated that even the lowest dose of HMOs (0.3 g/day) in children and adults significantly increased immune-associated metabolites, including aromatic lactic acids (indole-3-lactic acid and 3-phenyllactic acid) and 2-hydroxyisocaproic acid, as well as metabolites associated with the gut–brain axis, such as *γ*-aminobutyric acid, 3-hydroxybutyric acid, and acetylcholine (28). Notably, these aromatic lactic acids have been implicated in immune regulation (58) and neural processes mediated through the aryl hydrocarbon receptor (59–62).

The supplementation of 2′-FL and LNnT in an AAF for up to 12 months resulted in a significant increase in fecal SCFAs (17), mirroring previously observed elevation in plasma SCFA concentrations. This included marked increases in acetate, propionate, and butyrate, with acetate levels remaining consistently high throughout the study. These changes reflect the metabolic activity of *Bifidobacteria*, contributing to a lowered colonic pH and creating a protective acidic environment against entero-pathogens. The findings align with SCFA profiles commonly observed in breastfed infants, further underscoring the metabolic benefits of HMOs in infant nutrition.

### Clinical evidence of mechanistic insights into HMO action

HMOs exert their health benefits through a range of complex and interconnected mechanisms, influencing immune function, metabolic regulation, gut health, microbial composition, and systemic physiological processes. While few clinical studies have comprehensively mapped the specific pathways of HMO activity, emerging research highlights several key mechanisms.

#### Immunomodulatory effects of HMOs

Among HMOs, 2′-FL, being the most widely studied, plays a significant role in immune development. Studies show that 2′-FL supplementations significantly reduce pro-inflammatory cytokines in the plasma, aligning the innate immune profiles of formula-fed infants more closely with those of breastfed infants (34, 43). This modulation is especially beneficial in managing conditions such as CMPA, which is associated with heightened inflammatory cytokine expression. Beyond innate immunity, 2′-FL supplementation influences adaptive immunity responses by reducing disparities in T lymphocyte proportions and enhancing apoptotic activity, particularly in CD8 + T cells (43). By promoting the clearance of activated T cells, HMOs also help downregulate inflammatory responses, fostering a more regulated immune environment and potentially alleviating or preventing allergic reactions associated with CMPA.

#### Infection resistance

**Figure 2:**
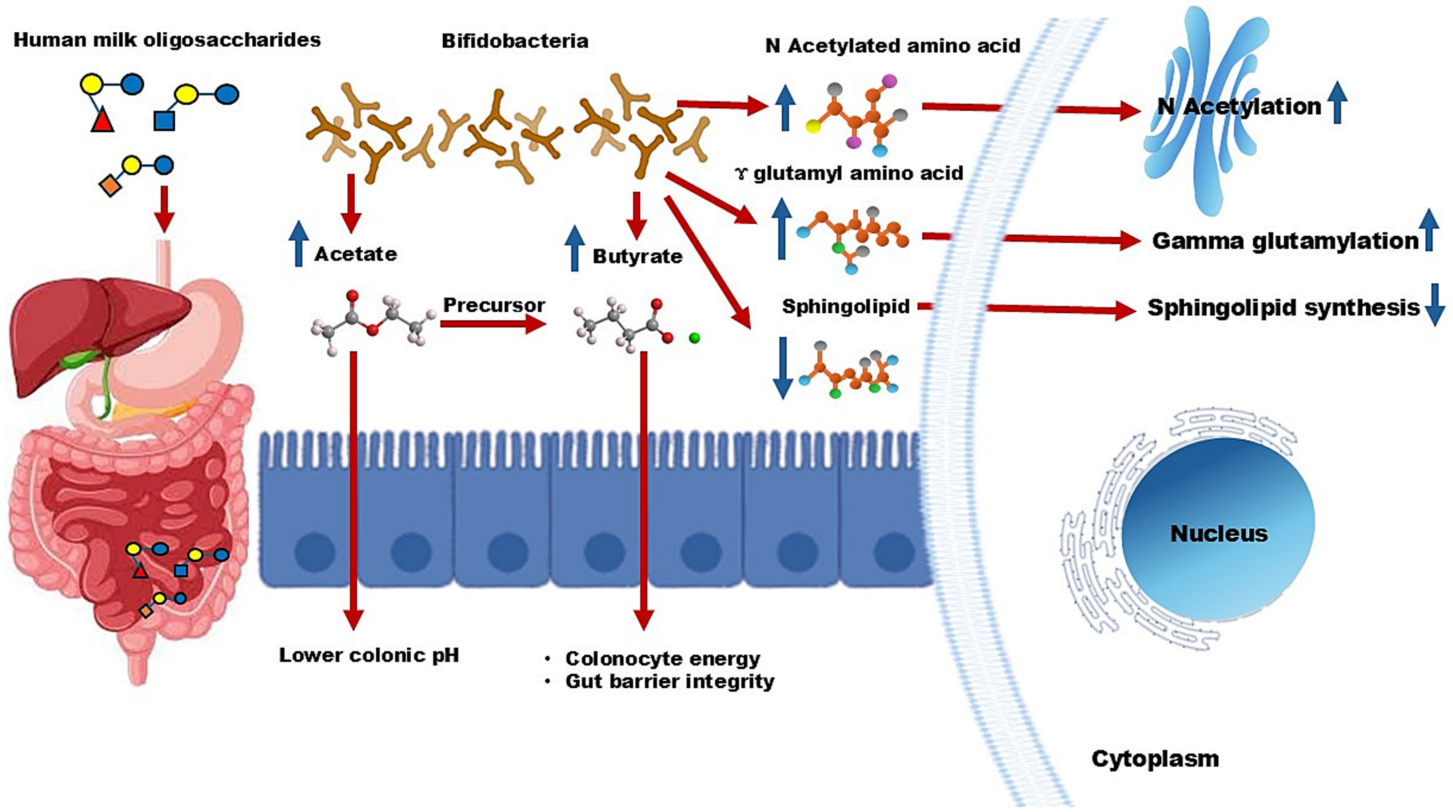
Mechanistic pathway of human milk oligosaccharides. *Bifidobacteria* in the colon utilize HMOs, leading to the production of acetate. This short-chain fatty acid lowers colonic pH and further works as a precursor for butyrate, which provides energy and maintains intestinal integrity. Additionally, HMOs boost levels of N-acetylated amino acids and gamma-glutamyl amino acids while reducing sphingolipid synthesis. These metabolic changes contribute to lower inflammation and enhance immune protection.

HMOs contribute to infection-resistance properties and gastrointestinal and metabolic health. Infants receiving HMO-supplemented formulas exhibit low incidences of URTIs and LRTIs and gastrointestinal disorders such as IBS (18, 26, 32, 40). The protective effects of HMOs are attributed to multiple mechanisms:

- Through gut microbiota modulation: HMOs promote beneficial gut bacteria, such as *Bifidobacterium, Bacteroides, and Lactobacillus,* thereby enhancing gut homeostasis (15, 42).
- Pathogen inhibition by acting as decoy receptors: HMOs prevent the adhesion of pathogens such as *Escherichia coli* and *Clostridium difficile*, reducing infection risks (35, 41).
- Through anti-inflammation: HMOs stimulate the synthesis of anti-inflammatory compounds, including N-acetyl amino acids and gamma-glutamyl amino acids, while reducing pro-inflammatory sphingolipids (Figure 2) (63).
- Innate immune enhancement: *In vitro* studies suggest that HMOs, such as 2′-FL and LNnT, can reduce respiratory syncytial virus (RSV) and influenza A viral loads by enhancing innate immune defense (64). However, these findings need to be confirmed through additional clinical studies.

#### Gut health and barrier integrity

Gastrointestinal disorders such as IBS, typically characterized by gut dysbiosis and compromised gut barrier integrity, are another area where HMOs show promise. Clinical studies reported that supplementation with 2′-FL and LNnT significantly improves both intestinal and systemic IBS symptoms (32, 40). These benefits are attributed, at least in part, to the prebiotic role of HMOs in promoting the growth of *Bifidobacteria*, which produce SCFAs such as acetate. Acetate lowers colonic pH, creating a favorable gut environment, and serves as a precursor for butyrate synthesis (Figure 2). Butyrate is essential for colonocyte energy metabolism and maintaining gut barrier integrity, further underscoring the role of HMOs in alleviating IBS symptoms.

#### Metabolic health benefits

Emerging evidence suggests that HMOs contribute to metabolic regulation by modulating gut microbiota and systemic inflammation. In line with this, a recent clinical trial was the first to examine the therapeutic effects of 3′-SL supplementation in patients with osteoarthritis, demonstrating that 200 or 600 mg/day of 3′-SL for 12 weeks led to significant pain relief and enhanced mobility (65). Besides, Canfora et al. (53) reported increased systemic butyrate levels in lean individuals and those with prediabetes or obesity after HMO supplementation. This elevation in butyrate was linked to improved metabolic markers, including reduced plasma-free fatty acids and systemic inflammation. These findings suggest that HMOs may play a beneficial role in preventing osteoarthritis, obesity, and associated metabolic disorders by modulating gut microbiota and promoting anti-inflammatory pathways. Clinical studies investigating the effects of manufactured HMOs on adults, with or without medical conditions, are shown in Table 4.

#### Gut–brain axis and neurodevelopment

Preclinical studies have demonstrated that SCFAs influence neuroactive pathways by crossing the blood–brain barrier, where they regulate brain energy metabolism, modulate inflammation, and affect neurotransmitter synthesis (66, 67). These mechanisms provide a plausible explanation for clinical findings that associate SCFA activity with indicators of appropriate neurodevelopment, as reflected in anthropometric measures such as head circumference. This link underscores the potential role of SCFAs in supporting early brain development and highlights their significance in shaping neurophysiological outcomes.

### Future perspectives

While considerable advancements have been made in understanding the benefits of HMOs, future research must address several limitations. One research gap is how HMOs influence cognitive development, which remains a key area requiring further exploration. Additionally, the metabolic fate of HMOs and the functional roles of their metabolites, particularly short-chain fatty acids (SCFAs), in peripheral organs involved in metabolic homeostasis—such as the liver, adipose tissue, and pancreas—also warrant further investigation. Furthermore, there has been limited focus on the mechanistic pathways in clinical investigations, with most studies emphasizing observation outcomes rather than the specific biological processes involved in reducing infections, limiting the ability to confirm causal relationships. Future clinical research should incorporate mechanistic endpoints to validate these effects. This is particularly important for comprehending how HMOs modulate immune responses, gut microbiota, and cognitive development. The current heterogeneity in study designs—such as variations in dosage, participant characteristics, and duration—further complicates the ability to draw consistent conclusions. To deepen our understanding, more randomized controlled trials (RCTs) are needed, especially those exploring long-term effects on immune modulation/inflammatory regulation, infection resistance/antimicrobial effects, gut microbiota modulation, gastrointestinal health, metabolic health, neurodevelopment, and the gut–brain axis, as well as the specific HMO structures and dosages required for clinical benefits. Addressing these gaps will be essential for developing targeted nutritional strategies to optimize human health outcomes.

Future directions should address broader applications in infant and adult nutrition, therapeutic potential in chronic diseases, and enhanced clinical research, including deeper exploration of the molecular pathways and systemic effects of HMOs in various physiological and pathological contexts. Large-scale, long-term trials evaluate the efficacy and safety of HMOs in diverse populations, including vulnerable groups like pre-term infants and elderly adults, and use of advanced technologies, such as genomics, metabolomics, and proteomics, to map HMO interactions and outcomes comprehensively. HMOs have the potential to redefine nutritional and therapeutic paradigms. Continued interdisciplinary research, technological innovation, and collaboration between academia, industry, and regulatory bodies will be critical in unlocking their full potential.
